# Identification of a Comprehensive Gene Co-Expression Network Associated with Autotetraploid Potato (*Solanum tuberosum* L.) Development Using WGCNA Analysis

**DOI:** 10.3390/genes14061162

**Published:** 2023-05-26

**Authors:** Zhimin Li, Juan Wang, Jiayin Wang

**Affiliations:** 1 School of Computer Science and Technology, Xi’an Jiaotong University, Xi’an 710049, China; zhiminli@stu.xjtu.edu.cn (Z.L.); juanwang2019@stu.xjtu.edu.cn (J.W.); 2 Shaanxi Engineering Research Center of Medical and Health Big Data, Xi’an Jiaotong University, Xi’an 710049, China

**Keywords:** autotetraploid, potato, RNA-seq, WGCNA analysis, hub genes

## Abstract

The formation and development of potato tissues and organs is a complex process regulated by a variety of genes and environmental factors. The regulatory mechanisms underlying the growth and development are still unclear. In this work, we aimed to explore the changes in gene expression patterns and genetic characteristics of potato tissues throughout different developmental stages. To achieve this, we used autotetraploid potato JC14 as an experimental subject to analyze the transcriptome of the root, stem, and leaf at the seedling, tuber formation, and tuber expansion stages. The results revealed thousands of differentially expressed genes, predominantly involved in defense response and carbohydrate metabolism according to KEGG pathway enrichment analysis. Weighted gene co-expression network analysis (WGCNA) revealed a total of 12 co-expressed gene modules, with 4 modules showing the highest correlation with potato stem development. By calculating the connectivity of genes within the module, hub genes were identified, and functional annotations were subsequently performed. A total of 40 hub genes from the four modules were identified, and their functions were found to be related to carbohydrate metabolism, defense response, and transcription factors. These findings provide important insights for further understanding of the molecular regulation and genetic mechanisms involved in potato tissue development.

## 1. Introduction

Potatoes (*Solanum tubulosum* L.) is the most consumed vegetable crop, the most common non-cereal food, and the fourth largest food crop following wheat, maize, and rice [1]. It is also an excellent source of energy, protein, vitamins, and minerals. In nature, there are various species of potatoes, such as 2N, 3N, 4N, 5N, and 6N polyploids [2]. Among them, the autotetraploid (4N polyploid) is the most widely cultivated commercially. The autotetraploid potato exhibits a high frequency of chromosome recombination and a low frequency of recessive gene expression, which can be preserved through asexual propagation. However, its genome is highly heterozygous, which poses challenges for genetic analysis and improvement. Therefore, it is important to investigate and identify molecular markers and key genes for the genetic development of autotetraploid potatoes. Next-generation sequencing has proven to be more efficient than traditional molecular biology techniques for mapping genes of interest [3,4]. In 2011, the International Potato Sequencing Team (PGSC) sequenced and assembled the genome of the heterozygous diploid potato RH (*S. tuberosum* group), providing a reference database for obtaining useful genetic information [1].

Weighted gene co-expression network analysis (WGCNA) can be used to identify gene modules based on the expression data obtained using gene chips or RNA-seq. It is a method that uses system biology to find co-expression networks and to reveal connections between genes and traits [4]. Moreover, WGCNA offers insights for screening signaling pathways and regulatory factors associated with target genes [5]. Currently, a number of publications have used WGCNA to study potato disease resistance and defense mechanisms. Yan et al. used different hormones to treat the late blight-resistant potato type SD20 and used WGCNA to analyze transcriptome data, resulting in the identification of nine defense genes that can be used as reliable functional verification targets [6]. Jing et al. used WGCNA analysis to find 13 TF-hub genes related to proline metabolism and salt stress resistance in potatoes [7]. Guo et al. analyzed gene expression in potato tissues under nitrogen-sufficient and nitrogen-deficient conditions and generated 23 modules from 116 differentially expressed hub genes associated with nitrogen metabolism, photosynthesis, and secondary metabolites using WGCNA [8]. Cao et al. identified several potato hub genes using WGCNA, including two genes involved in plant immune response regulation: *chitinases* and *flagellin-sensitive 2* [9]. Additionally, studies have also used WGCNA to explore the molecular mechanisms of plant growth and development in other species. Liu et al. created a gene co-expression network in rutabaga hypocotyl tuber, and WGCNA identified 59 co-expressed gene modules. Among these modules, two genes related to tuber growth, *Bra-FLOR1* and *Bra-CYP735A2*, were grouped in the same module. This module also included the genes involved in cell wall growth, secondary metabolism, auxin regulation, carbohydrate transport, and metabolism [10]. In contrast, only a limited number of studies have used the WGCNA method to investigate the growth and development of potatoes.

In this work, we aimed to explore the co-expression network of the genes involved in potato growth and development. Firstly, transcriptome sequencing was performed on the roots, stems, and leaves of the autotetraploid potato JC14 at three developmental stages, followed by differentially expressed gene analysis (DEG). Secondly, WGCNA was used to construct a gene co-expression network, and then the growth- and development-related gene modules were examined. Thirdly, KEGG and GO analyses were performed to identify the major metabolic pathways related to the target gene modules and potential gene functions. The key genes in each of the modules were then revealed based on the gene connectivity in the corresponding network. Finally, real-time quantitative PCR (RT-qPCR) was used to validate the expression pattern of these key genes during the growth and development of *S. tubulosum* L. This work constructed a comprehensive and dynamic co-expression network of the genes involved in potato growth and development. By elucidating the spatiotemporal expression of genes and the regulation mechanism of traits at different developmental stages, this research established a theoretical foundation for exploring the molecular mechanisms involved in the regulation of potato growth and development. Furthermore, it provides new insight and resources for autotetraploid potato breeding.

## 2. Materials and Methods

### 2.1. Potato Culture and Sampling

From 2022 to 2023, the potato genotype JC14 was cultivated in a greenhouse with natural light at Nankou Pilot Base, Chinese Academy of Agricultural Sciences, Beijing (longitude 116.408490; latitude 40.154860). The cultivation and administration processes were conducted in accordance with standard production procedures. Three biological duplicates of potato roots, stems, and leaves were collected during the seedling, tuber formation, and tuber expansion stages. Following collection, materials were cleaned in sterile water, immediately frozen in liquid nitrogen, and then stored at −80 °C for later total RNA extraction.

### 2.2. RNA Extraction, Illumina Sequencing, and Data Analysis

The total RNA of 27 samples was extracted using the TIANGEN plant RNA Extraction Kit (item number: DP762-T1) (TIANGEN, Beijing, China). The Nano Photometer^®^ spectrophotometer (IMPLEN, Westlake Village, USA) was used to assess the purity of the extracted total RNA. The Agilent 2100 RNA Nano 6000 Assay Kit and the Agilent 2100 Bioanalyzer (Agilent Technologies, Santa Clara, USA) were used to evaluate the concentration and integrity of the total RNA samples. RNA integrity RIN >= 7 indicates that the quality assessment was successful. Subsequently, 1–3 μg of total RNA from each sample was used to construct the transcriptome-sequencing library using the VAHTS Universal V6 RNA-seq Library Prep Kit from Illumina (No. NR604-01/02) (Vazyme, Nanjing, China). Briefly, mRNA was enriched from the total RNA using magnetic beads containing Oligo (dT), and the enriched mRNA was further tattered into short fragments. Random hexamers were used to synthesize single-strand cDNA, and then RNase H, dNTPs, buffer, and DNA polymerase I were added to produce double-stranded cDNA. The product was purified using the QiaQuick PCR Kit, and the purified cDNA was treated with end repair, A-tail addition, and sequencing adapter ligation, followed by fragment size selection. Finally, PCR was carried out to complete the cDNA library construction, and Qubit 3.0 was used for the preliminary quantification of the library. Bio-RAD KIT iQ SYBR GRN was then used to accurately quantify the diluted library concentration after diluting the library to a 1 ng/μL concentration. On the NovaSeq 6000 S4 platform, the Reagent kit V1.5 was used for clustering and sequencing, and 150 bp pair-end reads were performed.

Sequencing quality was evaluated using FastQC [11]. Low-quality reads and adapter sequences were trimmed from the raw reads using Trimmomatic. After trimming, clean data were mapped to the potato reference genome through HISAT2 [12]. The reference genome was obtained from the Spud DB Potato Genomics Resource (http://spuddb.uga.edu/rh_potato_download.shtml, accessed on 21 December 2022). The gene expression level was measured with FPKM using StringTie [13] and differential expression was analyzed using the DESeq R package [14]. A DEG was defined if the fold change was >2, and the q-value was <0.05 in this study.

### 2.3. Weighted Co-Expressed Gene Network Construction

The co-expressed gene network was created using the WGCNA package of the R software by following the instruction on the WGCNA website. [4]. Cluster analysis was performed using the Hclust function included in R, and samples that showed low correlation or could not be clustered in the dendrogram were removed. Then, using the parameter verbose = 5, the pick-soft threshold function in the WGCNA package was applied to calculate the soft threshold. When the fitting curve was close to 0.9 for the first time, the threshold parameter β was determined. The adjacency matrix was calculated using the adjacency function based on the value of β. The matrix was then transformed into a topological overlap matrix (TOM), and the gene connectivity network was constructed using the TOM similarity function. The Hclust function was used again to cluster TOM, and similar modules in the cluster were merged using the mergeCloseModules function. To calculate the signed eigengene-based connectivity, the signedKME function was used with parameters corFun = “cor”, and corOptions = “use = *p*”. A red and green color picture was produced using plotMat with the parameters nrgcols = 30 and cex.main = 2 for each module. Larger entries are indicated by an increasing intensity of red, whereas smaller entries are represented by an increasing intensity of green. To identify the correlation between modules and samples, the cor function was used to collect the matrix with the parameter use = “*p*”. The *p*-value between the correlation matrix and samples was calculated using the corPvalueStudent function. The module similarity threshold was set to 0.25, the expression threshold was set to 2, and the minimum number of genes in a module was set to 30 in this study.

### 2.4. The Selection of Key Modules and Enrichment Analysis

To investigate the gene modules involved in potato growth and development, correlation coefficients between genes in each module and various samples were calculated. The target gene module was determined based on a correlation coefficient greater than 0.5. Blast2Go was used to annotate DEGs in the module [15]. Gene ontology (GO) entries, such as molecular functions, cellular components, and biological processes, were also retrieved using Blast2Go. The pathway enrichment analysis of DEGs within the module was performed using the KEGG database [16].

### 2.5. Hub-Gene Screening and Functional Analysis

The intra-module gene significance, edges, and nodes of all DEGs selected from the four target modules were calculated using the WGCNA R package [4] (Table S1). The results were then transferred to the Cytoscape 3.9.1 software for gene network analysis and mapping [17]. DEGs with high connectivity in the module were chosen as candidate hub genes. The top 10 candidate hub genes in each module were selected as the hub genes after ranking based on their connectivity levels. The Swiss-Prot database [18] was used for homology annotation.

### 2.6. RT-qPCR Verification of Hub-Gene Expression

To validate the RNA-seq data, 20 genes were subjected to RT-qPCR to assess changes in relative expression levels, and the R package was used to calculate and plot the association between expression levels and FPKM. The expression levels of 40 candidate hub genes were also measured using RT-qPCR. All PCR primers were designed using Primer Premier 5.0 (Table S2). Potato stems from the seedling, tuber formation, and tuber expansion stages were used as raw materials. Hub-gene expression analysis was performed on the LightCycler480 system (Roche, Rotkreuz, Switzerland), with a total reaction volume of 10 µL, including 5 µL 2X SG Fast qPCR Master Mix (Roche, Rotkreuz, Switzerland), 0.2 µL primer (10 µM), 1.0 µL template, and 3.6 µL ddH2O. The expression level was calculated using the 2^−∆∆^ CT method, and *β-actin* was used as the reference gene [19]. The histograms were plotted using the Seaborn package in Python. Each sample had three biological duplicates, and significance and standard deviation were determined using an independent *t*-test with the Statannot package (ns: *p* > 0.05; *: *p* < 0.05, **: *p* < 0.01; ***: *p* < 0.001; ****: *p* < 0.0001).

## 3. Results

### 3.1. Genome-Wide Transcriptome Analysis

**Figure 1:**
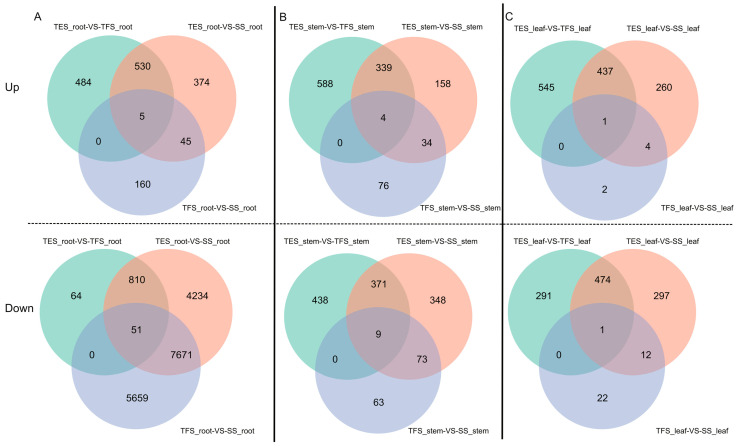
Venn diagram of regulated DEGs in different tissues of potatoes at different developmental stages. Different developmental stages: seedling stage, tuber formation stage, and tuber expansion stage: (**A**) (up and down) Venn diagram of potato roots DEGs of TES-VS-TFS, TES-VS-SS, and TFS-VS-SS different developmental stages; (**B**) (up and down) Venn diagram of potato stems DEGs of TES-VS-TFS, TES-VS-SS, and TFS-VS-SS different developmental stages; (**C**) (up and down) Venn diagram of leaves DEGs of TES-VS-TFS, TES-VS-SS, and TFS-VS-SS different developmental stages. SS: seedling stage; TFS: tuber formation stage; TES: tuber expansion stage; up: up-regulation; down: down-regulation.

With three biological duplicates per sample, RNA-seq was carried out on roots, stems, and leaves at three different developmental stages, yielding 176.36 GB of clean reads. Sequencing data for each sample were greater than 5.37 GB, the percentage of Q30 bases was higher than 91.89%, and the GC content ranged from 42.77% to 45.87% (Table S3). The read alignment of the samples ranged from 66.47% and 79.37% after mapping to the potato (RH89-039-16) reference genome [20] (Table S3). Between the seedling and tuber formation stages, the seedling and tuber expansion stages, and tuber formation and tuber expansion stages, 209, 953, and 1018 differentially up-regulated genes and 13,380, 12,765, and 924 down-regulated genes were identified in potato roots, respectively (Figure 1A). In stems, 113, 534, and 930 up-regulated genes and 144, 800, and 817 down-regulated genes were found, respectively (Figure 1B). In leaves, 7, 702, and 983 up-regulated genes and 34, 783, and 765 down-regulated genes were identified, respectively (Figure 1C).

### 3.2. Weighted Gene Co-Expression Network Construction and Module Identification

**Figure 2:**
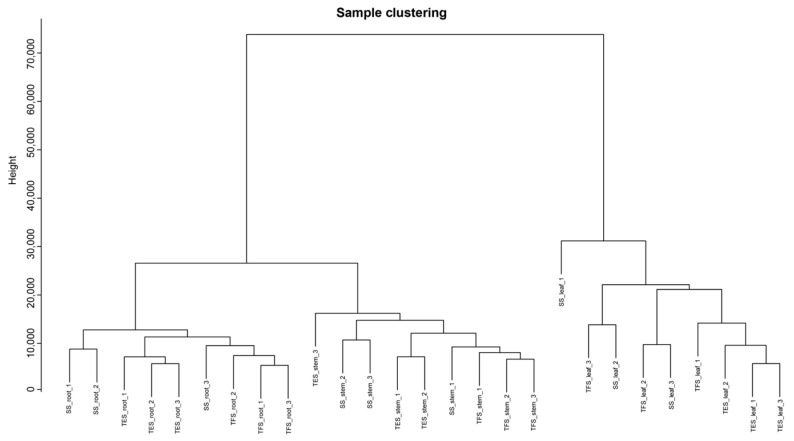
Phylogenetic dendrogram of all 27 samples. SS: seedling stage; TFS: tuber formation stage; TES: tuber expansion stage.

**Figure 3:**
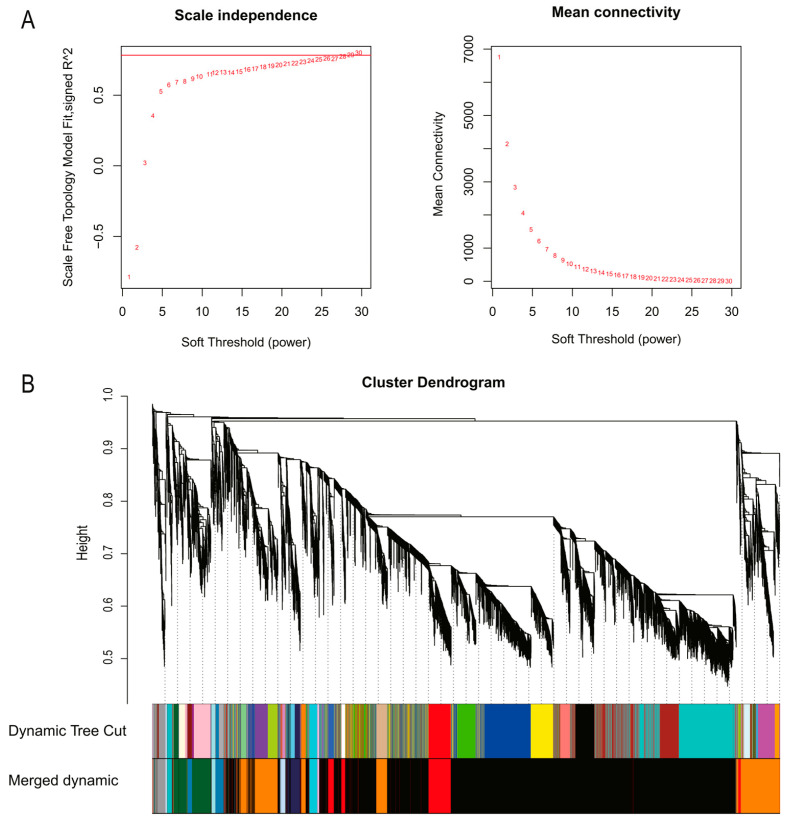
Soft-threshold determination for gene co-expression networks and module detection: (**A**) determination of the soft threshold; (**B**) detection of modules using the dendrogram.

The weighted gene co-expression network was constructed using a total of 14,475 genes. All samples underwent cluster analysis, and a correlation coefficient was obtained for each sample’s expression level. The results showed that all 27 samples were clustered, and no outliers were found (Figure 2). After calculating the weight values, the soft-threshold parameter β was determined to be β = 28 (Figure 3A). Then, genes with high correlation were allocated to the same module, and modules with similar expressions were merged using the dynamic tree-cut method. Different branches of the cluster tree represent different gene co-expression modules. To distinguish different modules, a color scheme was applied, and each module was given a color as its name. The result revealed 12 gene co-expressed modules with a total of 1546 genes (Figure 3B). Notably, 8 of the 12 modules (black, cyan, dark green, dark orange, grey 60, midnight blue, red, and steel blue) had 150 genes. The plum-1 module, on the other hand, had the lowest number of genes, with 41 genes (Table S4).

**Figure 4:**
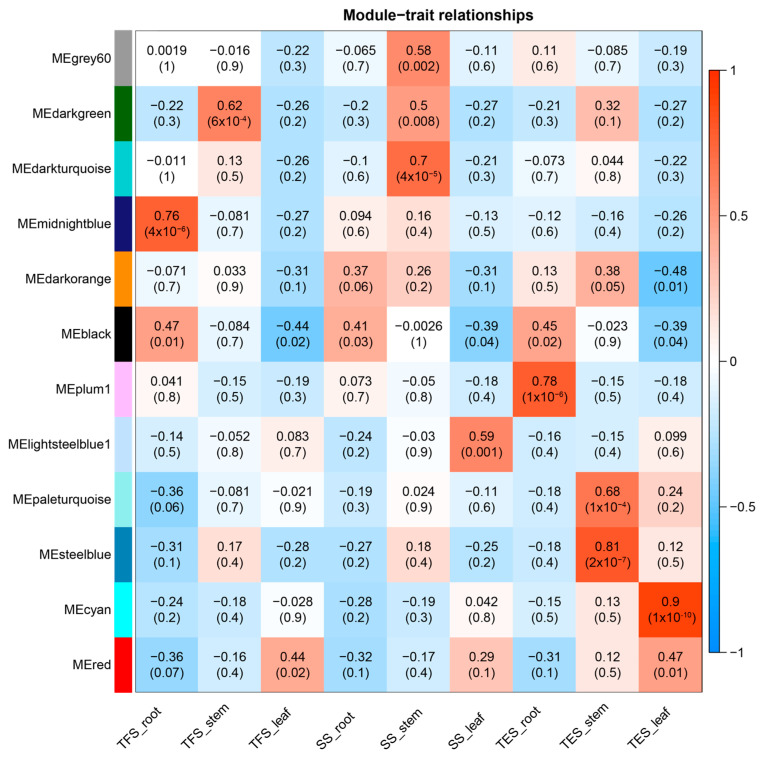
Correlation analysis between modules and traits revealed by Pearson correlation coefficient. Left-most co-expression modules are shown in different colors. The number in each box represents the correlation value between the module and the trait, and the numbers in brackets represent correlation *p*-values. SS: seedling stage; TFS: tuber formation stage; TES: tuber expansion stage.

Gene module and trait correlation coefficients were calculated, and the association between the modules and potato growth and development was examined. The Pearson correlation coefficient (r > 0.5) and the significance of the *p*-value (*p* < 0.05) were used as the criteria. The findings revealed that the midnight-blue module was significantly enriched in roots during tuber formation, whereas the plum-1 module was significantly enriched at the tuber expansion stage. In stems, only the dark-green module was significantly enriched at the tuber formation stage, and the steel-blue and pale-turquoise modules were significantly enriched at the tuber expansion stage. In contrast, the dark-turquoise module, as well as the grey-60 and dark-green modules, were significantly enriched at the seedling stage. Additionally, lightsteelblue1 was significantly enriched in leaves at the seedling stage, but cyan was significantly enriched at the tuber expansion stage. These results suggest that module enrichment in the same potato tissue differs between developmental stages and that enrichment is also very variable across different plant organs for the same developmental stage (Figure 4). Dark-turquoise, grey-60, dark-green, steel-blue, and pale-turquoise modules were chosen for further GO annotation and KEGG enrichment analysis because of their strong relationship to potato stem growth and development. However, due to the lack of any enriched pathway in the dark-turquoise module at the seedling stage, further analysis was not conducted for this module.

### 3.3. GO and KEGG Analysis of Key Modules in Different Tissues

In general, DEGs in seedling stems were primarily involved in defense-related metabolic pathways such as hormone signaling, MAPK signaling, and plant–pathogen interaction. During tuber formation, DEGs were found in amino and nucleotide sugar metabolism pathways, whereas in the tuber expansion stage, DEGs were found in growth-related pathways, including starch and sucrose metabolism, carbohydrate metabolism, and amino acid metabolism.

**Figure 5:**
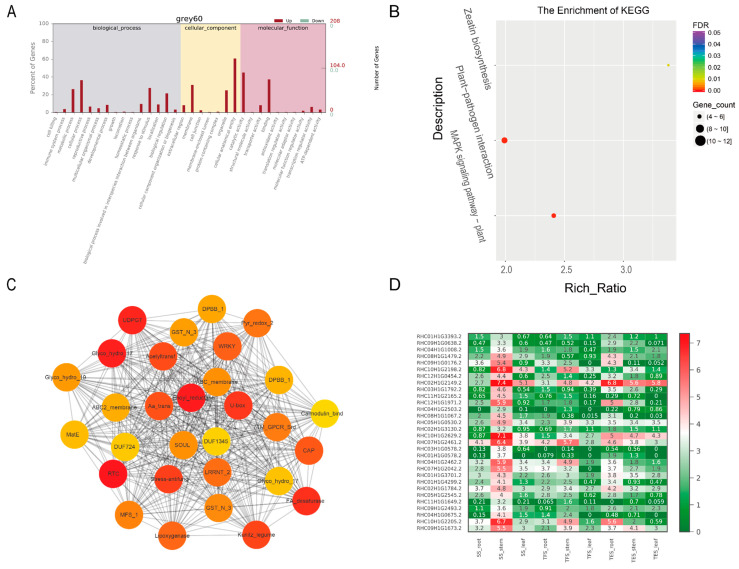
Analysis of genes of grey-60 module: (**A**) GO enrichment analysis for all genes of grey-60 module; (**B**) KEGG enrichment analysis for all genes of grey-60 module. The degree of enrichment in KEGG analysis increases as the color of the dots tends toward red. The number of genes enriched in the KEGG analysis was signified by the size of the dots, with larger dots containing a greater number of genes; (**C**) co-expression network for thirty hub genes in the grey-60 module. The top thirty hub genes had the highest connectivity, with the redder color representing higher connectivity; (**D**) heatmap of FPKM values (Log2(FPKM+1)) for thirty hub genes in root, stem, and leaf of potato SS, TFS, and TES stages. SS: seedling stage; TFS: tuber formation stage; TES: tuber expansion stage.

**Figure 6:**
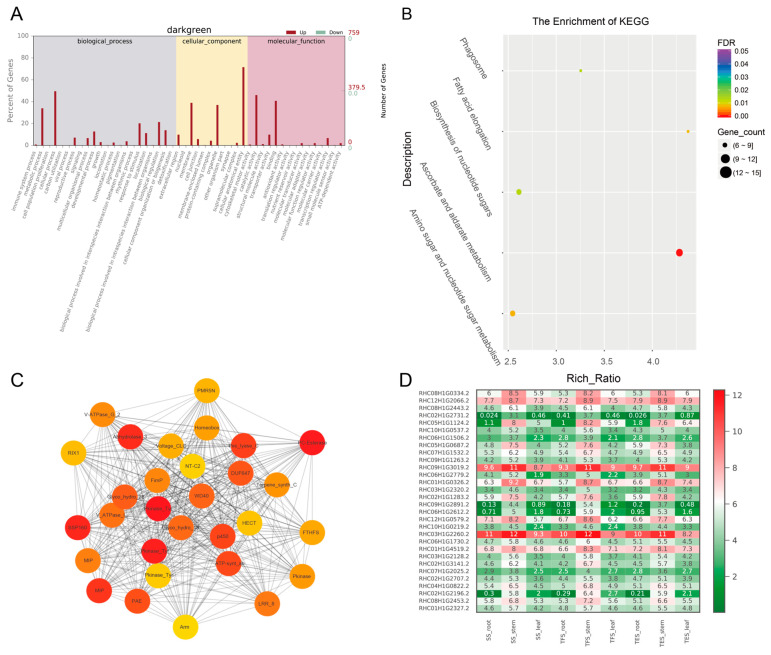
Analysis of genes from the dark-green module: (**A**) GO enrichment analysis for all genes of the dark-green module; (**B**) KEGG enrichment analysis for all genes of the dark-green module. The degree of enrichment in KEGG analysis increases as the color of the dots tends toward red. The number of genes enriched in the KEGG analysis is signified by the size of the dots, with larger dots containing a greater number of genes; (**C**) co-expression network for thirty hub genes in the dark-green module. The top thirty hub genes had the highest connectivity, with the redder color representing higher connectivity; (**D**) heatmap of FPKM values (Log2(FPKM+1)) for thirty hub genes in root, stem, and leaf of potato SS, TFS, and TES stages. SS: seedling stage; TFS: tuber formation stage; TES: tuber expansion stage.

**Figure 7:**
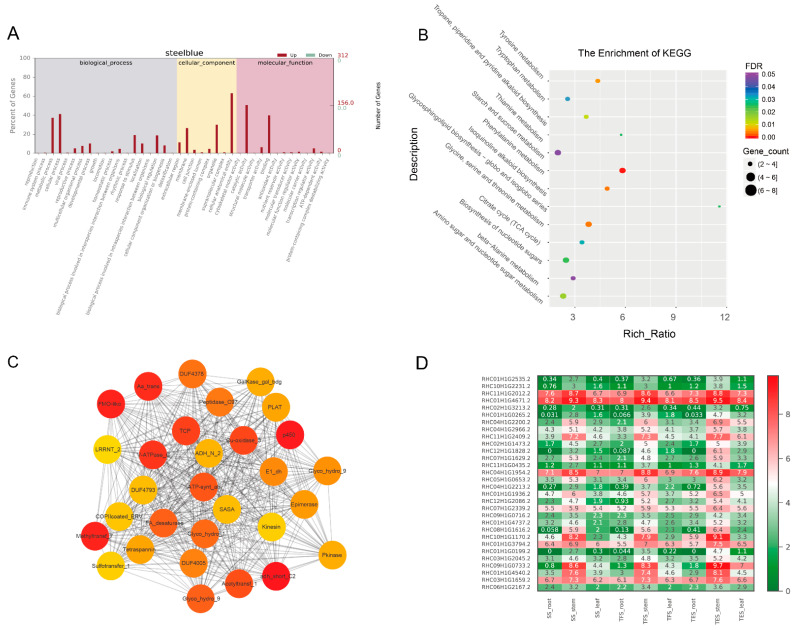
Analysis of genes from the steel-blue module: (**A**) GO enrichment analysis for all genes of the steel-blue module; (**B**) KEGG enrichment analysis for all genes of the steel-blue module. The degree of enrichment in KEGG analysis increases as the color of the dots tends toward red. The number of genes enriched in the KEGG analysis was signified by the size of the dots, with larger dots containing a greater number of genes; (**C**) co-expression network for thirty hub genes in the steel-blue module. The top thirty hub genes had the highest connectivity, with the redder color representing higher connectivity; (**D**) heatmap of FPKM values (Log2(FPKM+1)) for thirty hub genes in root, stem, and leaf of potato SS, TFS, and TES stages. SS: seedling stage; TFS: tuber formation stage; TES: tuber expansion stage.

**Figure 8:**
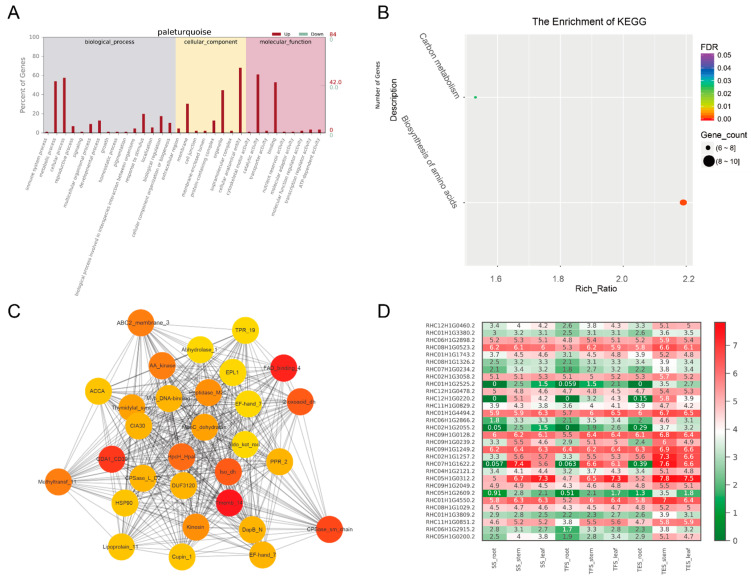
Analysis of genes from the pale-turquoise module: (**A**) GO enrichment analysis for all genes of the pale-turquoise module; (**B**) KEGG enrichment analysis for all genes of the pale-turquoise module. The degree of enrichment in KEGG analysis increases as the color of the dots tends toward red. The number of genes enriched in the KEGG analysis was signified by the size of the dots, with larger dots containing a greater number of genes; (**C**) co-expression network for thirty hub genes in the pale-turquoise module. The top thirty hub genes had the highest connectivity, with the redder color representing higher connectivity; (**D**) heatmap of FPKM values (Log2(FPKM+1)) for thirty hub genes in root, stem, and leaf of potato SS, TFS, and TES stages. SS: seedling stage; TFS: tuber formation stage; TES: tuber expansion stage.

Molecular function (MF), cellular component (CC), and biological process (BP) were the three GO groups for the four analyzed gene modules: grey 60, dark green, steel blue, and pale turquoise. The top GO terms in the MF group were binding, catalytic activity, and transporter activity; cells, cell parts, and organelles were found in the CC group; and the metabolic process, cellular process, and monomer process were included in the BP group (Figure 5A, Figure 6A, Figure 7A, Figure 8A and Table S5). According to the data, the grey-60 module was enriched in the olefin biosynthetic process (GO: 1900674), defense response (GO: 0006952), and chitin metabolism (GO: 0006030). The dark-green module was enriched in carbohydrate metabolism (GO: 0005975), cell growth (GO: 0016049), cell wall polysaccharide biosynthesis (GO: 0070592), acyltransferase activity (GO: 0016746), and methylammonium transmembrane transporter activity (GO: 0015200). The steel-blue module was enriched in carbohydrate metabolism (GO: 0005975), growth regulation (GO: 0040008), thiamine metabolism (GO: 0006772), hydrolase activity (GO: 0016787), and glucosidase activity (GO: 0015926). The pale-turquoise module was mainly enriched in amino acid metabolism (GO: 0006520), the primary metabolic process (GO: 0044238), steroid metabolism (GO: 0008202), carbon–nitrogen ligase activity (GO: 0016884), and acyltransferase activity (GO: 0016746). Overall, DEGs in the four target gene modules were found to be involved in the metabolic pathways linked to growth and development, such as carbohydrate metabolism, cell growth, and carbon–nitrogen ligase activity.

DEGs from the four target gene modules (grey 60, dark green, steel blue, and pale turquoise) were identified through KEGG analysis, and they were enriched in 3, 5, 13, and 2 pathways (FDR < 0.05), respectively (Figure 5B, Figure 6B, Figure 7B and Figure 8B). The grey-60 module was primarily involved in zeatin biosynthesis (map00908), MAPK signaling (map04016), and plant-pathogen interaction (map04626). The dark-green module was enriched in fatty acid elongation (map00062), phagosome (map04145), amino sugar and nucleotide sugar metabolism (map00520), nucleotide sugar biosynthesis (map01250), and ascorbate and aldarate metabolism (map00053). The steel-blue module was most abundant in the citrate cycle (TCA cycle) (map00020); serine, threonine, and glycine metabolism (map00260); tyrosine metabolism (map00350); phenylalanine metabolism (map00360); β-alanine metabolism (map00410); starch and sucrose metabolism (map00500); amino sugar and nucleotide sugar metabolism (map00520); thiamine metabolism (map00730); isoquinoline alkaloid biosynthesis (map00950); and tropane, piperidine and pyridine alkaloid biosynthesis (map00960). Carbon metabolism (map01200) and the biosynthesis of amino acids (map01230) were the two most prominent enriched pathways in the pale-turquoise module (Table S6).

### 3.4. Hub-Gene Screening and Functional Annotation

For co-expression network mapping, 30 genes with high connectivity from each of the four modules were chosen as candidate hub genes, and the red and orange colors in the network represent high and low connectivity, respectively (Figure 5C, Figure 6C, Figure 7C and Figure 8C). Based on the connectivity level, the top 10 ranked candidate hub genes from each of the four modules (40 genes in total) were selected as hub genes for further analysis (Table S7). The Swiss-Prot database was used for homology annotation on the 40 hub genes, and the majority of the genes were related to carbohydrate metabolism and plant growth and development. For example, the *reduced wall acetylation 3* (*RWA3*, RHC02H1G1413.2), *polygalacturonase* (*PG*, RHC08H1G2453.2), and *zeatin O-glucosyltransferase* (RHC05H1G02002.2) genes are involved in carbohydrate metabolism and cell division; the *glucanendo-1*,*3-β-glucosidase* (*GLU*, RHC10H1G2629.2) and *Cysteine-rich repeat secretory protein 55* (*CRRSP55*, RHC09H1G1673.2) genes are defense-response-related genes; transcription factors *MYB61* (RHC01H1G3797.2) and *PCF3* were found in transcription regulation; and *DUF642* is a recurrent conserved gene in the stems of many plants. Furthermore, a heat map was constructed using the RNA-seq data on hub-gene expression levels (Figure 5D, Figure 6D, Figure 7D, Figure 8D and Table S8). 

### 3.5. Changes in Gene Expression Levels of the Carbohydrate Metabolic Pathways

**Figure 9:**
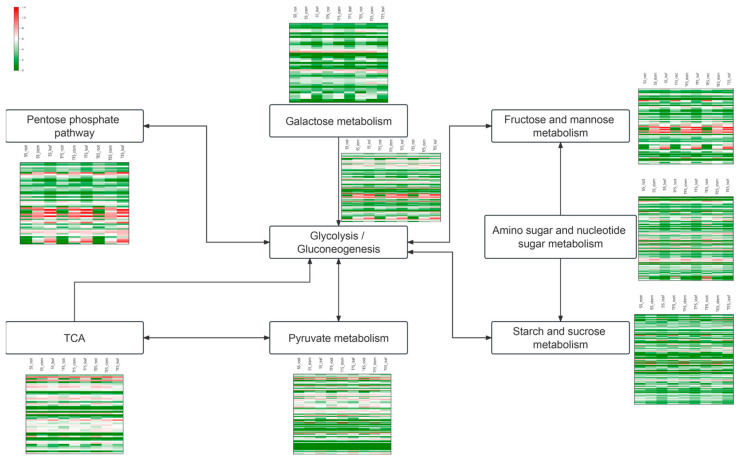
The expression of genes in root, stem, and leaf with different development stages of SS, TFS, and TES. Each row represents the expression level of a gene at different developmental stages, the color represents the Log_2_ (FPKM+1) value of different genes: red represents high expression, and green represents low expression. SS: seedling stage; TFS: tuber formation stage; TES: tuber expansion stage.

The carbohydrate metabolism contains eight pathways: the pentose phosphate pathway, galactose metabolism, fructose and mannose metabolism, glycolysis/gluconeogenesis, amino sugar and nucleotide sugar metabolism, TCA, pyruvate metabolism, and starch and sucrose metabolism. Among them, amino sugar and nucleoside sugar metabolism had the most genes (123), according to the statistical analysis. The TCA cycle-associated genes exhibited the highest expression during potato growth, indicating robust energy metabolism and fast growth and development in the roots, stems, and leaves of potatoes at the SS, TES, and TFS stages. The pentose-phase pathway, on the other hand, had a large number of highly expressed genes in stems and leaves, which suggests that it has a high metabolic level throughout the SS, TES, and TFS stages (Figure 9).

### 3.6. Validation of the Hub Genes Using RT-qPCR

**Figure 10:**
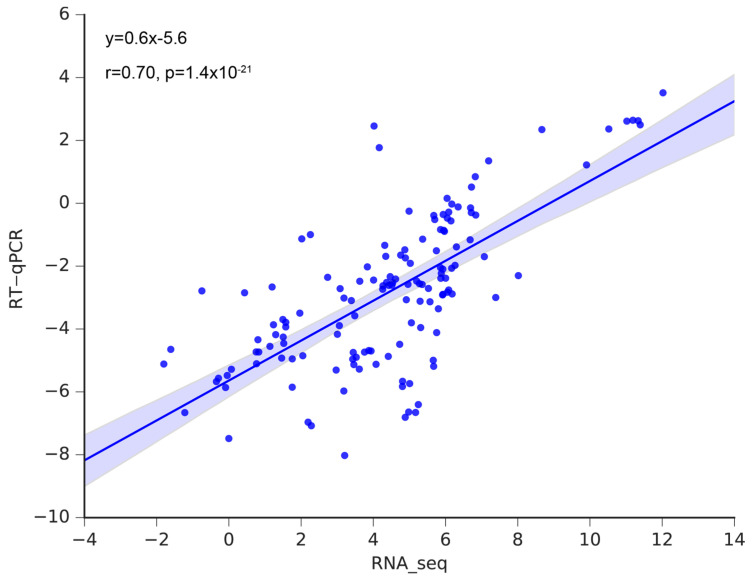
The correlation of expression levels determined using RNA-seq and RT-qPCR of the selected genes.

**Figure 11:**
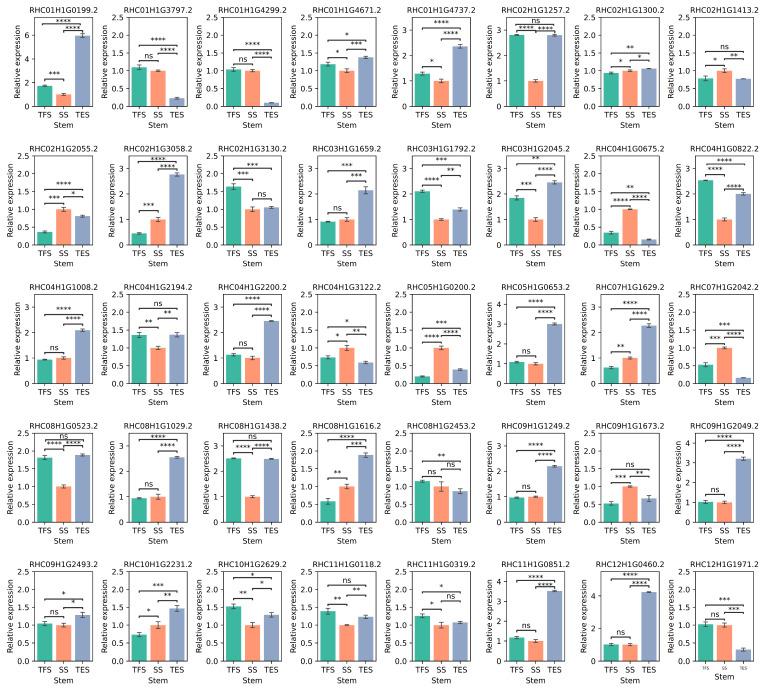
Relative expression of 40 hub genes in potato stems at seedling, tuber formation, and tuber expansion stages using RT-qPCR. *β-actin* was used as internal control. Three independent biological replicates were performed for each sample, and significance was determined using Mann–Whitney test included in the Statannot statistical package. The error bars represent standard deviations. (ns: *p* > 0.05; *: *p* < 0.05, **: *p* < 0.01; ***: *p* < 0.001; ****: *p* < 0.0001). SS: seedling stage; TFS: tuber formation stage; TES: tuber expansion stage.

To validate the RNA-seq data, 20 genes were selected for RT-qPCR. The results of RT-qPCR and RNA-seq had a strong correlation (R = 0.70), demonstrating the reliability of the transcriptome analysis (Figure 10). Furthermore, RT-qPCR was also employed to amplify the 40 hub genes. In accordance with RNA-seq data (Figure 5D, Figure 6D, Figure 7D, Figure 8D and Table S8), 14 hub genes were up-regulated during tuber formation and 27 during tuber expansion (Figure 10 and Table S8). This shows that these genes are important in the growth and development of potato stems. In comparison, 12 hub genes were down-regulated during tuber formation and 10 during tuber expansion (Figure 11 and Table S8), indicating that these genes may act as negative regulators of potato stem growth and development.

## 4. Discussion

Genetic analysis and improvement are obstructed by the high degree of genomic heterozygosity in cultivated cultivars of autotetraploid potatoes. Identifying key candidate genes for growth and development could lay the groundwork for future breeding. Previous studies employed the transcriptomic approach to uncover the genes implicated in the underlying mechanisms of potato cold resistance [21], disease resistance [22], and nitrogen utilization [8]. However, the mechanisms underlying potato growth and development are significantly more complex, with gene expression alteration occurring at various developmental stages in many metabolic pathways. Using transcriptome data from the roots, stems, and leaves of potatoes at different developmental stages, the WGCNA method was used in this study to establish a co-expression network of weighted genes associated with potato growth and development. As a result, 12 gene modules were identified, 4 of which exhibited high correlations with stem growth and development. A network diagram was then constructed using 30 candidate hub genes with high connectivity from each of the four modules (Figure 5C, Figure 6C, Figure 7C and Figure 8C), and the top 10 hub genes with the highest connectivity in each module were chosen for further investigation. Through carbohydrate metabolism, cell growth, defense response, and other related metabolic pathways, these hub genes may play essential roles in potato growth and development.

Carbohydrate is an important source of energy and is crucial for plant growth and development. Xylan is a type of heterogeneous polysaccharide found in plant cell walls, and the precise location and timing of its acetylation are important for plant growth and development. The RWA protein family regulates cell wall acetylation after xylan synthesis and is required for effective xylan binding to cellulose [23]. The overexpression of *RWA3* in *Dendrobium officinale* significantly increased polysaccharides’ acetylation level in seeds, leaves, and stems [24]. *PG* is involved in carbohydrate metabolism and is very important for cell wall metabolism and the maintenance of plant tissue morphology. Previous publications showed that *PG* is highly expressed in immature fruits and is associated with quality processes such as fruit ripening, flavor, and firmness [25,26]. In addition, the up-regulation of *PG* increases the tensile stress of *Catalpa bungei*, which promotes the development of high-quality wood [27]. In this study, *RWA3* and *PG* were highly expressed at all three stages, demonstrating the significance of their involvement in the growth and development stages of potato stems.

Auxins and cytokinins (CKs) are essential hormones that regulate plant growth and development. *O-glucosyltransferase*, a member of the O-glucosyltransferase superfamily [28], is a plant CK metabolic enzyme that catalyzes the reversible inactivation of O-glucosylation. It is important for plant CK homeostasis in vivo. *Zeatin O-glucosyltransferase*, another member of the superfamily, negatively regulates plant growth and development, and Dracaena’s long lifespan and slow growth may be due to its up-regulation [29]. In this study, *O-glucosyltransferase* expression was low in stems during tuber formation and high during tuber expansion, which may contribute to the slowdown of potato growth as development advances. However, the expression of *zeatin O-glucosyltransferase* remained low at all three developmental stages.

Proteins involved in pathogenesis are frequently activated under pathological conditions and are critical for disease resistance. GLU is a pathogenesis-related protein, and its expression is greatly promoted a few hours after *Phakopsora pachyrhizi* infection in soybeans. Soybean PEPPA-7431740 may stimulate immunity by inhibiting pathogen-associated molecular patterns such as interfering with the activity of 1,3-β-glucosidase [30]. The pesticide GLY-15 exhibits a strong antiviral effect against the tobacco mosaic virus in plants. It induces a stress response in plants, increasing the expression of 12 key genes, including *glucan-1*,*3-β-glucosidase* [31]. Other studies have demonstrated that *glucan endo-1*,*3-β-glucosidase* plays a role in plant responses to soil alkaline stress [32]. Notably, 1,3-β-glucan is a polysaccharide found in the cell walls of several phylogenetically distant organisms, including bacteria, fungi, plants, and microalgae, and it plays an important role in cell wall degradation [33]. CRRSP55 is a disease-related protein. MgMO237 promotes parasitism in Gramineae by interacting with the 1,3-β-glucan synthase component (OsGSC) and OsCRRSP55 to inhibit plant basal immunity during the late parasitic stage of nematode infection [34]. In this study, *GLU* and *CRRSP55* genes were expressed in stems throughout development (Figure 5A), suggesting that these genes may play an ongoing role in potato defense against pests and diseases.

Furthermore, *MYB61* and *PCF3* were two transcription factors selected from the module. Sugarcane *MYBs* play a crucial role in stem growth and stress responses [35]. Compared to ordinary wild soybean, seedlings of barren-tolerant wild soybean are more tolerant to phosphorus stress. It absorbs more phosphorus by increasing root length and *MYB61* is the key transcription factor for this resistance [36]. Rice *MYB61* may influence the efficiency of crop nitrogen utilization, which in turn affects rice growth and yield [37]. TCP (teosinte branched1/ccincinnata/proliferating cell factor) is a set of specific transcription factors that play an important role in plant growth and development, and *PCF3* is a member of the TCP family [38]. TCP transcription factors regulate leaf curvature, flower symmetry, and the synthesis of secondary metabolites. The majority of TCPs in *Ginkgo biloba* also respond to exogenous hormones, including ABA, SA, and MeJA [39]. In this study, *MYB61* expression was higher in stems during seedling and tuber formation and decreased during tuber expansion, suggesting that *MYB61* may be involved in drought and low phosphorus stress responses as well as nitrogen fixation in early development. The expression of *PCF3* in the stem increased gradually as growth and development progressed and peaked during the tuber expansion stage, implying that *PCF3* may promote potato stem growth.

## 5. Conclusions

In this work, weighted gene co-expression networks of roots, stems, and leaves at different developmental stages were constructed, and 12 target modules were identified. Four modules were associated with stem growth and development. According to GO and KEGG analysis, DEGs were found mostly in defense-related pathways, including plant and pathogen interaction, hormone signaling, MAPK, and growth-related pathways such as starch and sucrose metabolism, carbohydrate metabolism, and amino acid metabolism. Carbohydrate metabolism-related genes *RWA3* and *PG* were found to be highly expressed at all three stages, indicating their importance in stem growth and development. The limited expression of *zeatin O-glucosyltransferase* in stems during tuber formation and its high expression during tuber expansion may contribute to the potato’s slow growth rate during development. *GLU* and *CRRSP55* genes were expressed in stems at all three developmental stages, indicating that they continue to play a role in potato defense against pests and diseases. Furthermore, *MYB61* and *PCF3* were two transcription factors discovered in the target module, they regulate a number of downstream target genes and affect the growth of potatoes. The findings of this study will contribute to a better understanding of the molecular mechanisms of potato growth and development and provide new genetic resources for future autotetraploid potato breeding.

## Data Availability

The transcriptome data were uploaded to the NCBI database, and the accession number of the GEO database is SUB12997058.

## Supplementary Materials

The following supporting information can be downloaded at: https://www.mdpi.com/article/10.3390/genes14061162/s1, Table S1: Cytoscape input values of four target modules; Table S2: Primers used in this study; Table S3: Quality of evaluation of sequencing data; Table S4: Distribution of gene number in co-expression modules; Table S5: GO enrichment analysis of four target modules; Table S6: KEGG analysis of four target modules; Table S7: Functional annotations of hub genes; Table S8: Candidate hub-gene expression pattern by transcription measured.
